# Myelography in the Age of MRI: Why We Do It, and How We Do It

**DOI:** 10.1155/2011/329017

**Published:** 2011-03-06

**Authors:** Christoph Ozdoba, Jan Gralla, Alexander Rieke, Ralph Binggeli, Gerhard Schroth

**Affiliations:** ^1^University Institute of Diagnostic and Interventional Neuroradiology, Inselspital, University of Bern, Freiburgstrasse 4, 3010 Bern, Switzerland; ^2^Department of Neurosurgery, Inselspital, University of Bern, 3010 Bern, Switzerland

## Abstract

Myelography is a nearly ninety-year-old method that has undergone a steady development from the introduction of water-soluble contrast agents to CT myelography. Since the introduction of magnetic resonance imaging into clinical routine in the mid-1980s, the role of myelography seemed to be constantly less important in spinal diagnostics, but it remains a method that is probably even superior to MRI for special clinical issues. This paper briefly summarizes the historical development of myelography, describes the technique, and discusses current indications like the detection of CSF leaks or cervical root avulsion.

## 1. Introduction

The method that we know as “myelography” was first described by Sicard and Forestier [1] in 1921; by the end of the 1920s, it had become an established technique [2, 3]. In 1944, iodized oil (Lipiodol) was replaced by Iophendylate (Pantopaque) [4] as contrast agent for intrathecal application, but still, the procedure remained elaborate: the contrast agent that was applied by an intrathecal injection had to be withdrawn by suction at the end of the procedure, and the contrast agent itself was not free of adverse reactions [5, 6]. Yet, for decades myelography was the only diagnostic method that allowed to obtain information about soft-tissue structures in the spinal canal. Disc herniation, narrowing of the dural sac due to hemorrhage or tumor as well as expansion by intramedullary tumor, and nerve root compression that were not visible on conventional X-ray could be visualized.

In the seventies and eighties, the introduction of computed tomography and water-soluble nonionic contrast agents made the procedure easier to perform, safer and diagnostically more precise. Myelo-CT was first published in 1976 by Di Chiro and Schellinger [7], and it soon became a standard procedure.

Then, MR imaging found its way into clinical routine, and over a period of just some years, it made myelography look obsolete. A search for “myelography” in combination with “computed tomography” and/or “magnetic resonance” on PubMed yielded the results listed in Table 1 for the last six decades; according to these data, myelography's best years were obviously over at the end of the 1980s. It seemed that the method was on the same way that pneumencephalography had gone two decades before: from clinical routine into the archives of the history of medicine. Gradually, however, radiologists and clinicians realized that MR, although superior in many aspects, could not answer all questions related to spinal pathology.

Today, myelography is still established as a safe method [8] for some special clinical issues. The aim of this brief paper is to share our experience—which is based on about 6,000 myelographies in the last 17 years—regarding procedural aspects and instrumentation and to discuss the indications where myelography has remained the method of choice even in the early 21st century.

## 2. Myelography: How Do We Do It?

**Figure 1:**
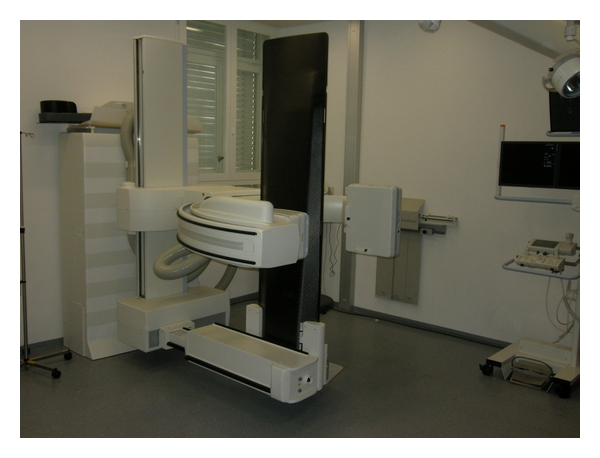
The myelography workplace. The table is tiltable by more than 90° so that a head-down position can be achieved.

**Figure 2:**
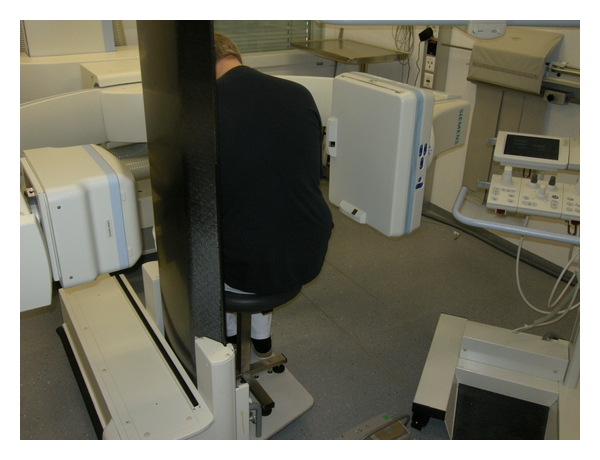
Volunteer demonstrating the patient position for the lumbar tap.

In many cases, patients scheduled for a myelography already have previous imaging studies; these are examined by the performing physician before the procedure to study the individual anatomy (e.g., scoliosis, Baastrup) and to select the most appropriate level for the puncture. The procedure should be performed with the lowest possible radiation exposure; this requires state-of-the-art fluoroscopic equipment. We use a Siemens Artis Multi-Purpose system (Siemens Medical Systems, Erlangen, Germany) with a fully tiltable patient couch (Figure 1). The puncture is performed with the patient in an upright position, that is, sitting on a specially designed chair; patients are instructed to form “a cat's arched back” (Figure 2).

This is not in accordance with the guidelines for myelography jointly defined by the American College of Radiology and the American Society of Neuroradiology [9] that suggest a prone position; in our experience, however, sitting for just some minutes (with support, if necessary) is possible for most patients, and it vastly simplifies the lumbar puncture. The rounded back makes sure that the spinous processes in the lumbar spine are distracted as far as possible. 

We usually perform the spinal tap at lumbar level 2/3; this ensures that we do not accidentally puncture the conus, and it is just above the clinically most often affected segments so that we avoid a puncture into a herniated disc. Standard for the puncture is a 20 G (0.9 mm) 90 mm Quincke needle (Pic Indolor, Artsana S.p.A., Grandate, Italy).

In routine procedures, 5–10 ml CSF are taken for laboratory studies. Then, the contrast agent (Iopamiro (lumbar: 200 and 300, 10 ml each; cervical: 300, 20 ml), Bracco, Milan, Italy) is injected under fluoroscopic control. This allows to immediately identify and correct accidental injections into the epidural space and to check whether the contrast flow is obstructed. A picture with the needle *in situ* is taken for documentation, then, the needle is removed. The patient couch is rotated to a horizontal position with the patient still in a “sitting” position on the chair.

**Figure 3:**
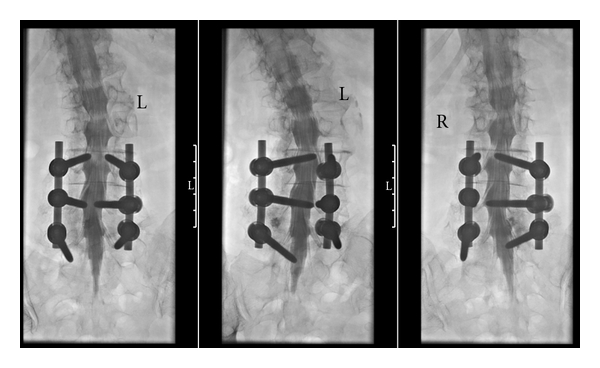
Standard projections in prone position. From left to right: a.p., about 25° left and right to show the lumbar nerve roots. Taking these images under fluoroscopic control makes sure that even with stabilizing material on three levels the roots are visible from their origin to the foramen.

For lumbar myelography, contrast filling should reach up to the thoracic level D10 so that the conus is included. The special chair is then removed, the patient turned in the prone position on the stomach and dural sac and root filling are documented in strict a.p.-view and by rotating the C-arch so that the lumbar roots are optimally visualized, that is, about 25° lateral in each direction (Figure 3).

**Figure 4:**
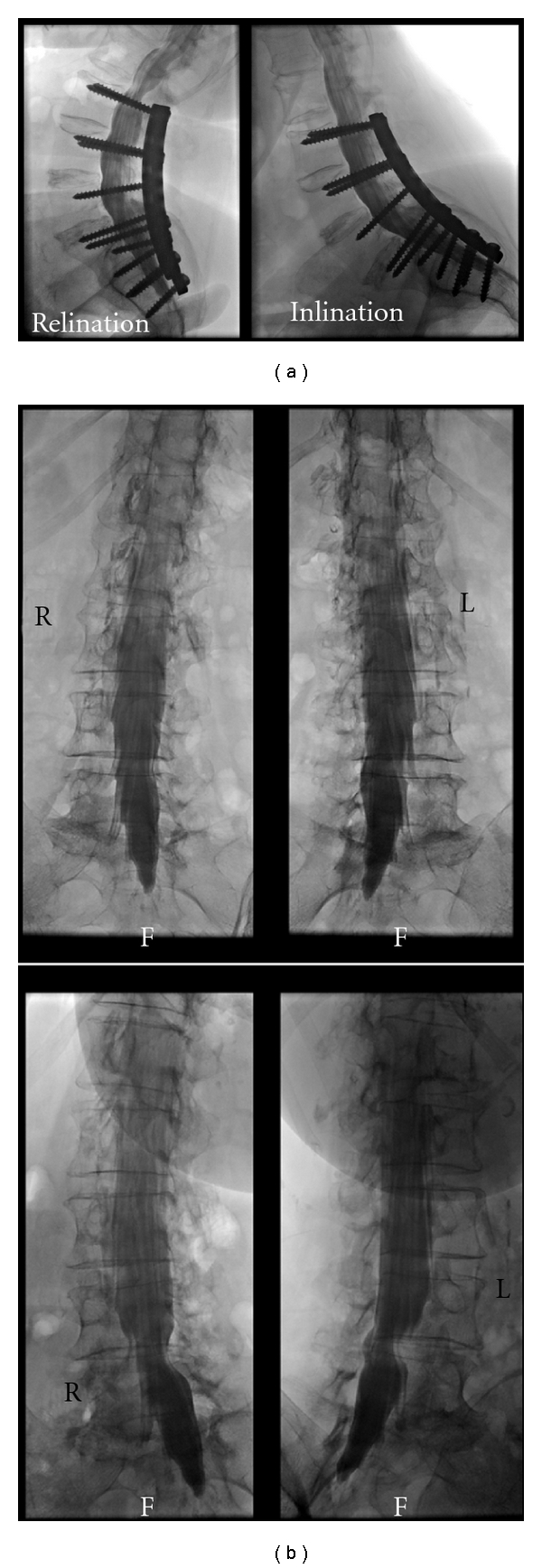
Diagnostic value of additional upright/functional views. (a) Extension (left) shows marked narrowing of the sagittal dural sac diameter directly above the stabilization. The finding in flexed position (right) is normal. This information cannot be obtained in the prone position alone. (b) Oblique views, top: prone position, bottom: patient standing upright. Shortening of the left L4 root and compression of the left L5 origin are only visible in the upright position.

Then, the table is tilted so that the patient gets into an upright (standing) position. The a.p. and oblique shots are repeated and functional pictures in flexion and extension are taken. The ACR/ASNR guidelines do not mention these additional projections; in our experience, however, they may be the diagnostically most relevant of the study (Figure 4). The whole procedure does not take more than five minutes for an experienced team.

**Figure 5:**
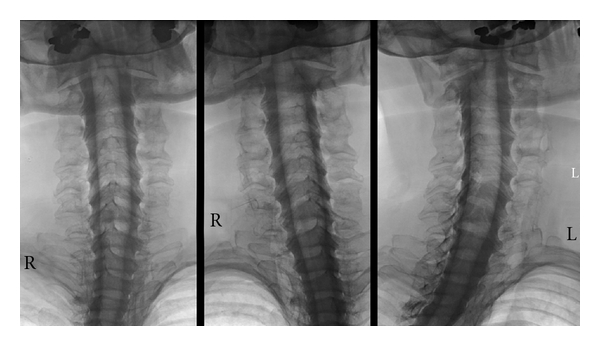
Cervical myelography (prone position). With the patient's head reclined, there is sufficient time to acquire images that show the cervical nerve roots in high detail without losing contrast. (Standard projections as Figure 3).

For cervical myelography which we only perform ascending via lumbar puncture for safety reasons, it is important to instruct the patient to keep the head reclined during the contrast injection, that is, while still lying on the side. This ensures that the contrast agent does not enter the intracranial CSF spaces. It is usually necessary to tilt the patient couch head down by 10–15° to pass the thoracic spine. Again, the upwards contrast flow is followed by fluoroscopy. When contrast has reached the lower part of the cervical spine, the patient is turned on the stomach. This rotation should be done by the team, not by the patient himself, to avoid excessive motion that might drive the contrast column unwantedly far upwards. The patient's head must remain reclined. With the patient in the prone position lying on the stomach, a.p. and oblique views are taken (Figure 5).

## 3. Myelography: When Do We Do It?

**Figure 6:**
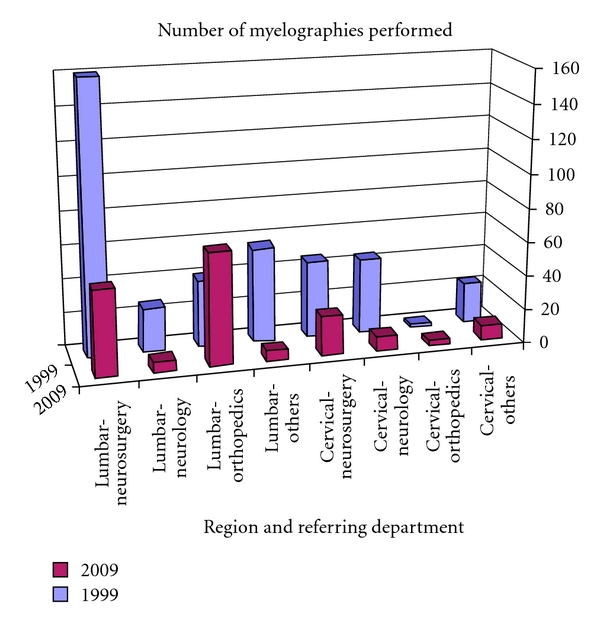
Development of myelography exams at the authors' institution 1999–2009.

The majority of patients at our institution are referred for myelography by orthopedic surgeons and neurosurgeons.  Table 2 and Figure 6 show that today, the total number of these procedures is less than 45% of what it was ten years ago.

Aside from patients where MR imaging is not possible for safety reasons (e.g., pacemaker), severe image quality degradation due to metallic implants, claustrophobia, or in cases where kyphoscoliosis makes image acquisition and interpretation extremely difficult, however, there are still indications for myelography as an independent diagnostic tool.

MRI seems to be the ideal tool for spinal imaging as it has some obvious advantages over myelography/myelo-CT: no lumbar puncture, no X-ray exposition, no intrathecal contrast agents, excellent soft-tissue contrast.

Modern MRI, however, is not automatically superior to “old-fashioned” myelography: Bartynski and Lin [10] have shown that nerve root compression in the lateral recess is underestimated by MRI in nearly 30% of surgically confirmed cases compared to only 5 to 7% in myelography. While a study published in 2005 [11] saw no difference in the diagnostic and predictive value of myelography, myelo-CT and MRI in cases of severe spinal stenosis, a recent Japanese study [12] found myelography with CT myelography “more reliable and reproducible than MRI” when deciding on which levels decompressive lumbar surgery should be performed. Furthermore, and especially important in cases where surgery is discussed, MRI tends to underestimate the width of the spinal canal and the foramina, thereby making spinal stenosis appear more severe than myelography/myelo-CT [13, 14].

**Figure 7:**
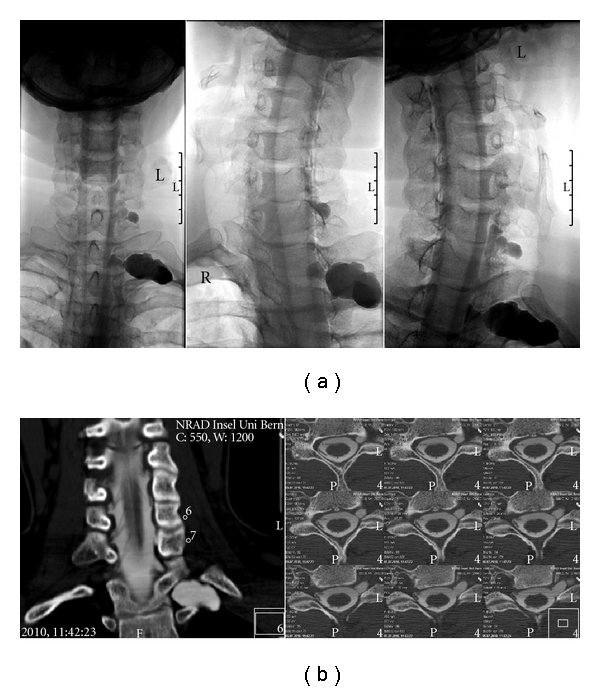
Cervical root avulsion after motorcycle accident. (a) Myelography shows traumatic pseudoceles C7-D1. Rootlets are not discernible. (b) Thin-section (1.25 mm) myelo-CT and reformatted coronal images clearly show complete avulsion of ventral and dorsal rootlets.

A special clinical situation that requires detailed high-resolution imaging is cervical root avulsion. The typical meningocele is easily identified in any imaging modality, but an older study [15] indicates that myelography is superior to MRI in delineating the ventral and dorsal rootlets with an accuracy of 85% for CT myelography compared to 58% for MRI in relation to intraoperative findings. More recent studies reported an accuracy of 88% for MRI [16] and a sensitivity of 100% for CT with coronal and oblique coronal reformatted views [17] so that further studies will be necessary to definitely decide which method is the most appropriate in this setting. We mostly use combined myelography and myelo-CT with good results (Figure 7).

**Figure 8:**
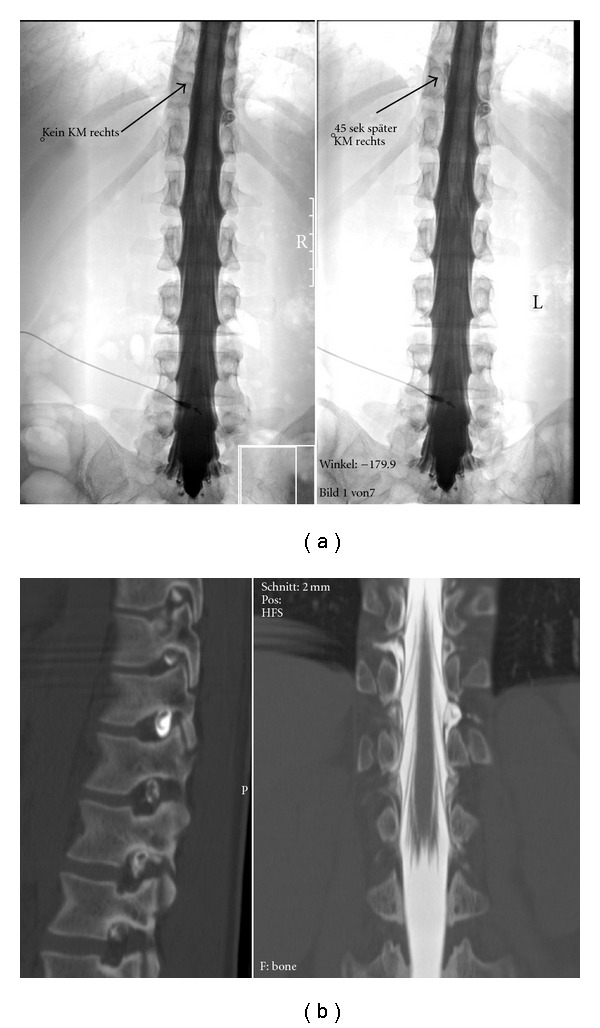
Spinal CSF leak causing subdural hematoma. (a) Left: contrast leakage to the left at the level of the D11 root. Right: 45 seconds later, contrast has flown around the dural sac and is exiting the spinal canal to the right. The dynamic series easily allows to study these flow dynamics and avoids misinterpretations. (b) Sagittal (left) and coronal (right) reformatted images from the subsequent myelo-CT show leakage in the left D11/12 foramen and contrast leakage to the right one segment above. This static study does not allow to exactly determine how contrast flows in and around the dural sac.

A condition that has recently gained some attention is chronic intracranial subdural hematoma due to a spinal CSF leak. Case reports [18, 19] show that MR imaging is clearly inferior to myelography in locating the location of the leak. We have made the same experience in some cases; the possibility to dynamically visualize and record the contrast flow makes myelography the method of choice in these cases (Figure 8).

## 4. Conclusion and Perspective

Myelography is no longer the gold standard in the diagnosis of disc herniation and root compression. It is, however, more than just a makeshift when MRI is not possible; myelography can provide valuable diagnostic information beyond MRI: the option to acquire dynamic imaging sequences, including positional changes of the patient, and the combination with CT that delivers undistorted images—even with metallic implants—with high spatial and contrast resolution ensure that myelography will remain in the portfolio of neuroradiologic diagnostic tools. 

The recently introduced technique of “positional MRI” that allows to examine patients in an upright position including functional (flexion, extension, rotation) views in a vertical-bore low-field MR scanner [20, 21] has not gained widespread acceptance yet; the future will show whether this technique can actually replace functional myelography.

As myelography is on the way to become a “special procedure" for selected cases, it becomes even more important that neuroradiologists world-wide make sure that training in myelography remains included in residents' curricula so that experience with this procedure remains available for the next generation of physicians.

## Figures and Tables

**Table 1:** Results of a PubMed search for “myelography” alone and in combination with “computed tomography” and/or “magnetic resonance”.

Publication date from—to	Citations containing “myelography”	Citations containing “myelography” and “computed tomography”	Citations containing “myelography” and “magnetic resonance”	Citations containing “myelography” and “computed tomography” and “magnetic resonance”
1950–1959	202	—	—	—
1960–1969	1051	—	—	—
1970–1979	2183	81	—	—
1980–1989	3226	1385	363	243
1990–1999	1902	896	865	507
2000–2009	987	191	579	121

Source: U.S. National Library of Medicine/National Institutes of Health (http://www.ncbi.nlm.nih.gov/pubmed).

**Table 2:** Myelographies in the authors' institution by region and referring department: comparison between 1999 and 2009.

1999	Neurosurgery	Neurology	Orthopedics	Others	Total
Cervical	45	44	1	28	118
Lumbar	160	26	40	56	282

Total.					**400**

2009	Neurosurgery	Neurology	Orthopedics	Others	Total

Cervical	23	8	3	9	43
Lumbar	50	6	66	6	128

Total.					**171**
